# TRPV1 antagonists attenuate antigen-provoked cough in ovalbumin sensitized guinea pigs

**DOI:** 10.1186/1745-9974-2-10

**Published:** 2006-12-15

**Authors:** Robbie L McLeod, Xiomara Fernandez, Craig C Correll, Tara P Phelps, Yanlin Jia, Xin Wang, John A Hey

**Affiliations:** 1 Peripheral and Pulmonary Neurobiology Schering-Plough Research Institute, Kenilworth, NJ, 07033-0539, USA

## Abstract

We examined the molecular pharmacology and in vivo effects of a TRPV1 receptor antagonist, N-(4-Tertiarybutylphenyl)-4(3-cholorphyridin-2-yl)-tetrahydro-pyrazine1(2H) – carboxamide (BCTC) on the guinea pig TRPV1 cation channel. BCTC antagonized capsaicin-induced activation and PMA-mediated activation of guinea pig TRPV1 with IC_50 _values of 12.2 ± 5.2 nM, and 0.85 ± 0.10 nM, respectively. In addition, BCTC (100 nM) completely blocked the ability of heterologously expressed gpTRPV1 to respond to decreases in pH. Thus, BCTC is able to block polymodal activation of gpTRPV1. Furthermore, in nodose ganglia cells, capsaicin induced Ca^2+ ^influx through TRPV1 channel was inhibited via BCTC in a concentration dependent manner. In in vivo studies capsaicin (10 – 300 μM) delivered by aerosol to the pulmonary system of non-sensitized guinea pigs produced an increase in cough frequency. In these studies, the tussigenic effects of capsaicin (300 μM) were blocked in a dose dependent fashion when BCTC (0.01–3.0 mg/kg, i.p.) was administered 30 minutes before challenge. The high dose of BCTC (3.0 mg/kg, i.p) produced a maximum inhibition of capsaicin-induced cough of 65%. We also studied the effects of BCTC (0.03 and 3.0) when administered 60 minutes before capsaicin. Under these conditions, BCTC (3.0 mg/kg, i.p) produced a maximum decrease in capsaicin-induced cough of 31%. In ovalbumin passively sensitized guinea pigs, we found that BCTC (1 and 3 mg/kg, i.p.) attenuated antigen ovalbumin (0.3%) cough responses by 27% and 60%, respectively. We conclude that TRPV1 channel activation may play role in cough mediated by antigen in sensitized guinea pigs. Our results supports increasing evidence that TRPV1 may play a role in the generation of the cough response.

## Background

The vanilloid receptor (TRPV1) is a member of a distinct subgroup of transient receptor potential (TRP) family of ion channels [1]. The neuronally expressed TRPV1 is a non-selective, Ca^2+ ^preferring, cation channel. The TRPV1 channel is activated by a number of different stimuli including heat, acid certain arachidonic acid derivatives and direct phosphorylation via PKC [2-5]. Moreover, there is also evidence that various inflammatory mediators such as ATP, bradykinin, NGF or PGE_2 _may indirectly lead to the activation of the TRPV1 channel via activation of their respective receptors [6-9]. Current data suggests that receptor activation may lead to TRPV1 gating by either generation of arachidonate via BK_2 _or through the activation of PKC by P2Y_1 _[6-10]. These findings suggest that TRPV1 may have a central role in inflammatory nociception.

Within recent years, pulmonary researchers have shown an interest in TRPV1 and the possible role of this receptor in respiratory diseases [11]. TRPV1 has been linked to playing significant role in the genesis of cough. Indeed, cough is arguably the most common symptom associated with pulmonary diseases, such as asthma, COPD and the common cold [12-14]. The evidence for this linkage between TRPV1 and cough is supported by several observations. (1) TRPV1 receptors are found on sensory airway nerves that are important in the cough reflex [15-17]. (2) Isolated pulmonary vagal afferent nerves are responsive to TRPV1 stimulation and (3) TRPV1 agonists, such as capsaicin, elicit cough in animals and man [18-21]. (4) Furthermore, sensitivity of capsaicin-induced cough responses following upper respiratory tract infection and in airway inflammatory diseases such as asthma and COPD, are increased relative to control responses [22,23]. Nonetheless, it is important to point out that although cough can be provoked by aerosolized capsaicin to the airways, the significance of TRPV1 receptors in cough associated with pulmonary diseases remains to be fully elucidated.

N-(4-Tertiarybutylphenyl)-4(3-cholorphyridin-2-yl)tetrahydropyrazine-1(2H)-carbox-amide (BCTC) is a highly potent and selective TRPV1 antagonist [24]. This new pharmacological tool has been used to block TRPV1 responses in inflammatory and neuropathic pain models [25]. Presently we studied the TRPV1 antagonist activity of BCTC in HEK293^OFF ^cells stably-expressing gpTRPV1 and in isolated guinea pig nodose ganglia. As our primary goal, we sought to utilize BCTC to examine the role of TRPV1 receptors in antigen-induced cough in ovalbumin sensitized guinea pigs. We found that BCTC attenuated cough in a model of antigen-provoked cough.

## Materials and methods

### Animal care and use

These studies were performed in accordance to the NIH GUIDE TO THE CARE AND USE OF LABORATORY ANIMALS and the Animal Welfare Act in an AAALAC-accredited program.

### RNA isolation, cloning and expression of guinea pig TRPV1

Male Hartley Short Hair guinea pigs (350 – 400 g) were euthanized with CO_2_, and the nodose ganglia were dissected and flash-frozen in liquid nitrogen prior to total RNA isolation. Total RNA was prepared from nodose ganglia using the Ambion Totally RNA kit (Ambion, Austin, TX, USA) according to the manufacturer's instructions. First strand cDNA synthesis was carried out and used to carry out PCR reactions using an Ex Taq Kit (Pan Vera, Madison, WI, U.S.A.). Multiple primers were designed based upon the published guinea pig sequence (GenBank #AJ492922) and used to generate short fragments for establishment of a consensus sequence. The resulting full length sequence (GenBank #AY729017) was used to clone a full length gpTRPV1 sequence from primary tissue. The following primers were used to clone out gpTRPV1 in two fragments P1:atgaagaaacgggctagtgtgg, P2: gccagagccagtggtgtgaaccccttc, P3:gaaggggttcacaccactggctctggc, P4: tcacttctcccctggaactgtcggactc. The resulting fragments were used to create a full length gpTRPV1 cDNA sequence which was subcloned between the NotI and EcoRV sites of the pTRE2hyg vector (BD Biosciences, Clontech, Palo Alto, CA) for sequence confirmation and Tet-promoter controlled expression of gpTRPV1. Stably-transfected HEK293Tet^OFF ^cells expressing gpTRPV1 under control of the Tet-promoter were maintained in MEM medium (supplemented with 10% Tet System Approved FBS/penicillin/streptomycin/L-glutamine/geneticin G418, all from Invitrogen, Caisbad, CA) at 37°C and 5% CO_2 _in a humidified atmosphere.

### Molecular pharmacology

Analysis of gpTRPV1 activity was carried out using FLIPR as described previously [26]. Briefly, HEK293^OFF ^cells stably-expressing gpTRPV1 were plated in black clear-bottomed 96-well poly-lysine plates (BD Biosciences) at a concentration of 40,000 cells per well in 200 μl of media in the absence of doxycycline to allow for expression. The plates were incubated for two days at 37°C and 5% CO_2 _to allow for optimal expression of TRPV1. The cells were incubated in a buffer consisting of Hank's Balanced Salt Solution (HBSS) containing 10 mM HEPES pH 7.4, BSA 1%, and probenecid 2.5 mM with the addition of the calcium sensitive fluorescent dye Fluo-4AM (Molecular Probes, Eugene, OR) (4 μM) for 1 hour at 37°C. The cells were washed 3 times with the above buffer, which had been heated to 37°C. A total of 100 μl of buffer was placed in to each well and the plates were put in a 37°C incubator for an additional 30 minutes prior to assay. All compounds used in these studies were dissolved in dimethyl sulfoxide (DMSO) and vehicle alone (DMSO) was used as a control. The cells were then placed in a FLIPR (Molecular Devices, Sunnyvale CA) with a heated stage maintained at 37°C for monitoring changes in fluorescent signal upon addition of agonist. After addition of compound, change in fluorescence was monitored for a period of 5 min and maximal increase in fluorescent signal was noted. Antagonist was added to cells in a volume of 50 μl via the FLIPR and allowed to incubate for 6 minutes prior to addition of agonist. The change in fluorescence (max – min) upon addition of agonist was used to assess activation.

### Intracellular Ca^2+ ^concentration measurements in nodose ganglia cells

Male Hartley guinea pigs (600 – 700 g, Charles River, Bloomington, MA, USA) were euthanized with CO_2_. The nodose ganglia were removed under aseptic conditions and enzyme digested as previously described [17]. Briefly, the isolated ganglia were washed in Hank's buffer (Gibco, NY, USA) and then transferred to Hank's buffer containing collagenase (type IA, 1 mg • ml^-1^) for 45 min at 37°C in a water bath. The enzyme solution was aspirated from the tissues, after which they were rinsed with Hank's buffer and then incubated in Hank's buffer containing DNAse IV (0.1 mg • ml^-1^) for 15 min at 37°C in a water bath. Tissues were washed with Hank's buffer and subjected to gentle trituration using a Pasteur pipette. The resulting cell suspension was filtered through a sterile nylon mesh (Becton Dickinson Labware MA, USA) and plated into poly-lysine coated petri dishes (Becton Dickinson Labware MA, USA). Cells were incubated for 3 hrs at 37°C prior to the intracellular Ca^2+ ^measurements. Intracellular Ca^2+ ^concentrations in single nodose ganglia cells was measured in Hank's buffer using Attofluor digital ratiovision system (Atto Instrument, Maryland, USA). Briefly, cells were incubated with Fura-2 acetoxy methylestor (5 μg ml^-1^, Molecular Probes), a calcium sensitive fluorescence dye, in HBSS containing 0.4% bovine serum albumin (BSA) for 45 min at 37°C. The dye-loading solution was removed and the cells were washed three times with HBSS containing 0.4% BSA. Fluorescence in single cells was measured at a single emission wavelength (510 nm) with double excitatory wavelength (334 and 380 nm), using Attofluor digital ratiovision system. Intracellular Ca^2+ ^concentration was estimated by ratio of fluorescence at excitation wavelengths of 334 and 380 nm. Capsaicin responses were elicited by direct additions to the cell culture buffer during real-time recording

### Capsaicin-induced cough

All cough experiments were performed in conscious guinea pigs (Male Hartley, 400 – 500 g, Charles River, Bloomington, MA, USA) using methods described by Bolser et al., [20]. In the first experiment, the effect of graded concentrations of aerosolized capsaicin was examined on cough frequency. Overnight fasted guinea pigs were placed in a 12 × 14-inch chamber and exposed to aerosolized capsaicin (10 – 300 μM, for 4 min) produced by a Ultra-NeB 99 Devilbiss nebulizer (Somerset, PA) to elicit cough. Experiments were conducted in a parallel design where each animal was exposed only once to capsaicin. The number of coughs were detected by a microphone placed in the chamber and verified by a trained observer. The signal from the microphone was relayed to a polygraph that provided a record of the number of coughs. The antitussive activity of BCTC was determined against cough provoked by capsaicin (300 μM). In these studies, BCTC (0.01 – 10 mg/kg, i.p.) was given 30 minutes before capsaicin challenge. In a separate study, the cough suppressant effects of BCTC (0.03 and 3.0 mg/kg, i.p.) was studied at 1 hour after i.p. administration.

### Antigen-induced cough

Male Hartley guinea pigs (300 – 350 g, Charles River, Bloomington, MA, USA) were actively sensitized to ovalbumin over a 27 day regimen. On day 1, animals were administered ovalbumin (100 μg, i.p.) and aluminum hydroxide (200 mg, i.p.) suspended in 0.5 ml of water. On day 7, animals were administered an additional dose of ovalbumin (100 μg, i.p.). The animals were used 27 days after the initial ovalbumin dose when they weighed between 450 – 500 g. Allergic cough studies were performed in an exposure chamber similar to the one used to examine capsaicin-evoked cough responses. The concentration of ovalbumin (0.3%) used to elicit cough was selected based on studies by Bolser et al., [20]. BCTC (1 and 3 mg/kg, i.p.) was given 30 minutes before ovalbumin (0.3%). The activity of a second TRPV1 antagonist was also studied in these experiments, capsazepine (300 μM; 4 min aerosol) was given 4 minutes before antigen challenge.

### Statistics

Data from HEK293^OFF ^cells studies are presented as the percentage of the maximal response for each agonist. Calculation of IC_50 _values were determined using GraphPad Prism v3.02 (GraphPad Software, Inc.). Data from the cough studies are expressed as cough number due to either a capsaicin or a ovalbumin 4 minute exposure. Values displayed in the figures represent the MEAN ± SEM of 6–12 animals per group. Data were evaluated using a non parametric Kruskal Wallis in conjunction with a Mann Whitney U. Statistical significance was set at p < 0.05.

### Drugs

Capsaicin, capsazepine, and phorbol 12-myristate 13-acetate (PMA) were purchased from Sigma (St. Louis, MO, USA). N-(4-Tertiarybutylphenyl)-4(3-cholorphyridin-2-yl)tetrahydropyrazine1(2H)-carbox-amide (BCTC) was synthesized based on to published reports and was tested in all experiments as the free base (molecular weight 372.89) [24]. For molecular and in vtiro studies drugs were dissolved in dimethylsulfoxide (DMSO) and stored at -20.0 °C. The final concentration of DMSO was less than 0.1% (v/v) in these studies. For in vivo studies, capsaicin and capsazepine were dissolved in 10% ethanol and physiological saline (0.9%), respectively. BCTC was dissolved in warm (58°C) 45% (2-hydroxypropyl-) β-cyclodextrin.

## Results

### Intracellular Ca^2+ ^concentration measurements in HEK293^OFF ^cells

**Figure 1:**
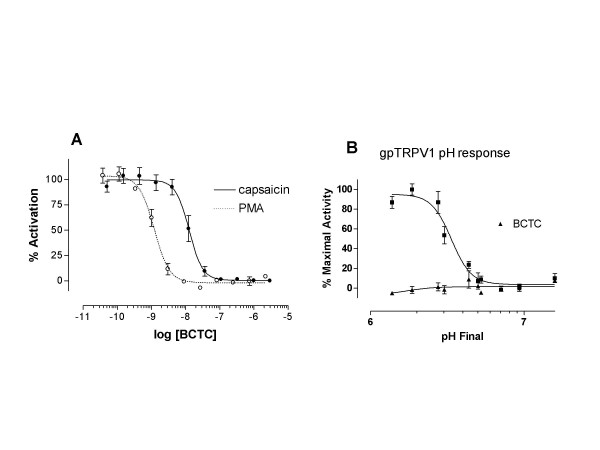
Inhibition of TRPV1 polymodal activation by BCTC in HEK293^OFF ^cells. Panel A shows that BCTC antagonizes capsaicin (10 nM) and PMA-mediated (50 nM) activation of gpTRPV1. Panel B shows that inclusion of 100 nM BCTC completely inhibits gpTRPV1 respond to decreases in pH. Data are presented as percent maximal response in the absence of inhibitor (A). Data shown are representative of at least three separate experiments.

The TRPV1 antagonist BCTC was tested for its ability to inhibit various modalities of guinea pig TRPV1 activation. BCTC dose-dependently inhibited capsaicin-induced activation and PMA-mediated activation of guinea pig TRPV1 with IC_50 _values of 12.2 ± 5.2 nM, and 0.85 ± 0.10 nM, respectively (see Figure 1A). The addition of 50 nM PMA to gpTRPV1 expressing cells which were pre-incubated with 1 μM Ro 31–8220, a PKC inhibitor, elicited no response (data not shown). Additionally, capsazepine was able to block both modes of TRPV1 activation with potencies relative to previously described results [27]. The inclusion of 100 nM BCTC completely blocked the ability of gpTRPV1 to respond to decreases in pH (see Figure 1B).

### Nodose ganglia

**Figure 2:**
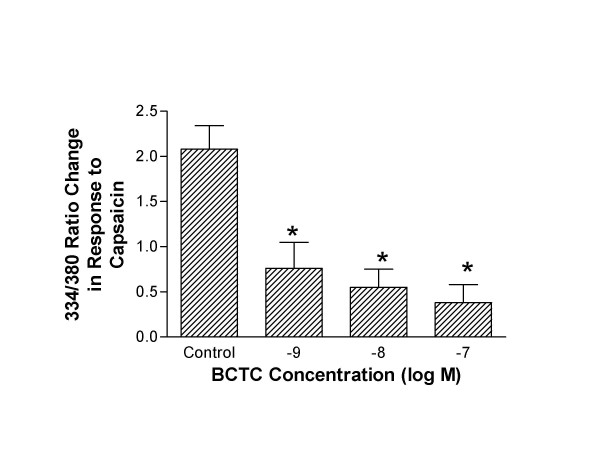
Intracellular Ca^2+ ^in response to capsaicin (0.1 μM) was measured in isolated guinea pig nodose ganglia neurons and expressed as 334/380 ratio change. When cells were preincubated with BCTC, capsaicin-induced Ca2+ response was inhibited in a concentration dependent manner. * p < 0.05 compared with control (n = 5–12).

Previously we have shown that capsaicin increases intracellular Ca^2+ ^in guinea pig nodose ganglia cell, in a concentration-dependent manner [28]. In the present study we evaluate the activity of BCTC against the increase in nodose intracellular Ca^2+ ^elicited by 0.1 μM capsaicin. The change in the 334/380 fluorescence ratios due to capsaicin (0.1 μM) was 2.08 ± 0.26. BCTC (1 × 10^-9 ^– 1 × 10^-7^M) significantly attenuated capsaicin-induced intracellular Ca^2+ ^responses in nodose ganglia cells (see Figure 2).

### Cough studies

**Figure 3:**
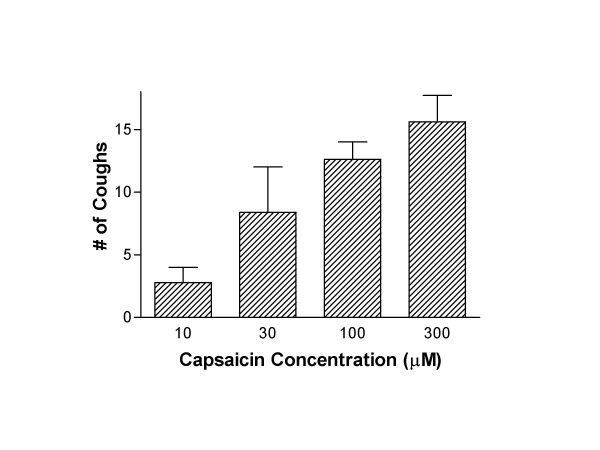
Tussigenic effects of capsaicin in non-sensitized conscious guinea pigs. Figures shows that aerosolized capsaicin (10 – 300 μM, 4 min exposure; n = 6–8 per treatment group) produces a dose-dependent increase in cough frequency in guinea pigs. The tussigenic response to a saline (which produced no coughing; n = 5) is not shown in the figure.

**Figure 4:**
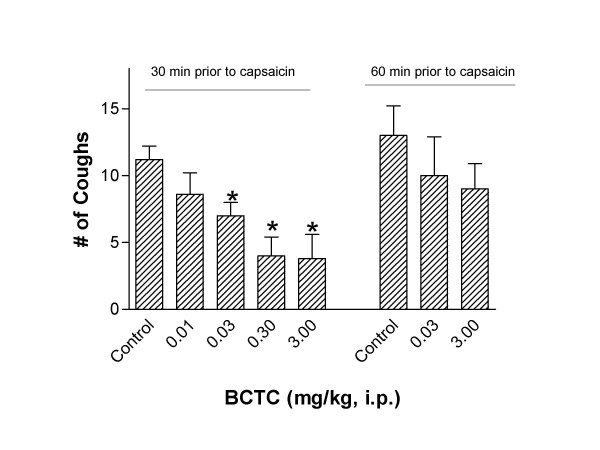
Effect of BCTC on capsaicin-induced cough in non-sensitized guinea pigs. Figure demonstrates the cough suppressant activity of BCTC (0.01 – 3.0 mg/kg, i.p.) administered at 30 and 60 minutes before capsaicin (300 μM) provocation. Each bar represents the Mean ± SEM of the number of coughs produced by a 4 min exposure to capsaicin. Control animals were guinea pigs that received oral vehicle instead of BCTC and were exposed to capsaicin provocation. (*p < 0.05 compared to control animals using a Kruskal-Wallis in conjunction with a Mann-Whitney-U; n = 8–9 per treatment group).

**Figure 5:**
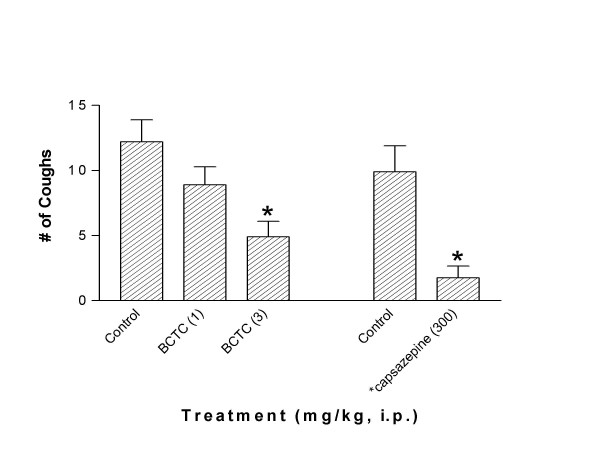
Effect of BCTC on cough responses elicited by antigen challenge in sensitized guinea pigs. BCTC (1 and 3 mg/kg, i.p.) blocked the increase in cough produced by antigen ovalbumin (0.3%) challenge. Also shown are the activities of a second TRPV1 antagonist (given by aerosol 4 min before antigen provocation*), capsazepine (300 μM) on allergic cough. Each bar represents the Mean ± SEM of the number of coughs produced by a 4 min exposure to capsaicin. (*p < 0.05 compared to controls (sensitized and administered vehicle) animals using a Kruskal-Wallis in conjunction with a Mann-Whitney-U; n = 9–16).

In non-sensitized naive animals, aerosolized exposure to capsaicin (10–300 μM) increased guinea pig cough frequency (see Figure 3). In follow-up studies we used the 300 μM concentration of capsaicin as the provocation dose to examine the cough suppressant activity of BCTC. Capsaicin (300 μM) produced 15.6 ± 2.1 coughs over a 4 minute exposure time (see Figure 4). Figure 4 shows that 30 minutes after i.p. administration BCTC (0.01–3.0 mg/kg, i.p.) dose dependently attenuated the increase in cough frequency provoked by capsaicin (300 μM). We found that the optimum experimental protocol for the BCTC cough studies was to give the drug i.p. 30 minutes before capsaicin, because by 60 minutes the cough suppressant activity of BCTC was significantly diminished (see Figure 4). Using the experimental design established in the capsaicin studies, BCTC (3 mg/kg, i.p.) was administered in sensitized guinea pigs 30 minutes before cough was provoked by ovalbumin. BCTC inhibited allergic cough by 60% (see Figure 5). Doses of BCTC greater than 3 mg/kg could not be tested because of solubility limitations of the drug. To confirm the antitussive actions of BCTC against antigen-induced cough, a structurally different TRPV1 antagonist was also studied. Similar to BCTC, aerosolized capsazepine (300 μM) blocked cough (-81%) elicited by ovalbumin (See Figure 5).

## Discussion

Recently, van den Worm et al., (2005) demonstrated that a TRPV1 receptor antagonist inhibits isolated allergen-induced tracheal contractions [29]. The objective of the present studies was to examine the role of TRPV1 receptors in an allergic "disease" cough model. To this end, we utilized the recently described TRPV1 antagonist, BCTC, as a pharmacological tool in our experiments. BCTC has been shown to inhibit rat TRPV1 channels. However, its effect on guinea pig TRPV1 has not been tested previously. Prior to advancing BCTC into in guinea pig in vitro and in vivo experiments, we first characterized the activity of this drug on guinea pig TRPV1 in HEK293^OFF ^cells that heterologously expressed cloned guinea pig TRPV1 receptor. We found the BCTC effectively antagonized the prototypical activity of the vanilloid receptor agonist, capsaicin. Additionally, BCTC abolished proton-mediated and antagonized PKC-phosphorylation-induced activation of TRPV1. The potency of BCTC against PMA-induced activation was significantly more potent than against capsaicin-driven activation. The mechanism behind this difference is unclear, however, we have observed that BCTC is more potent in antagonizing PMA-induced activation in other TRPV1 orthologues including human, mouse and rat [26].

Stimulation of a PKC phosphorylation pathway could link TRPV1 mediated pulmonary responses with the upstream activation of cell surface receptors such as the purinergic receptor P2Y_1_, bradykinin BK_2 _receptor, PAR2, histamine H1 receptor, or the nerve growth factor (NGF) receptor TrkA [6-8,30]. Indeed, recent results demonstrate that PAR2-mediated sensitization of TRPV1 enhances the overall cough reflex and, by utilizing specific inhibitors, this exaggerated response appears to be mediated via PAR2 -induced PKC and/or PKA activity. Therefore, our results suggest that BCTC may not only effectively antagonize the direct activation of TRPV1 receptors via small molecule but may also block the actions of inflammatory mediators (trypsin, bradykinin, histamine, e.g.) that may indirectly contribute to TRPV1 activation/sensitization, by stimulating PKC activity. Furthermore, our experiments also demonstrate that the antagonist activity of BCTC is observed at the level of the native TRPV1 receptor in guinea pig nodose ganglia. The present BCTC data are consistent with previous finding showing that capsaicin-induced Ca^2+ ^responses in isolated guinea-pig nodose ganglia cells are blocked by the TRPV1 antagonist, capsazepine [17]. Nodose ganglia cells relay sensory impulses into the CNS from a variety of visceral organs, including the pulmonary system. Moreover, nodose ganglia (and jugular ganglia) contain the cell bodies of airway afferent sensory nerves that are involved in the cough reflex. Thus, our in vitro studies indicate, at least in part, a peripheral pharmacological action for BCTC on C-fibers nerves which are known to express TRPV1 receptors. Activity of BCTC on respiratory associated C-fibers likely contributes to the antitussive action of this drug observed in our cough models.

Chemical irritants such as capsaicin and citric acid are often used to elicit cough in experimental models. Both capsaicin and citric acid directly activate TRPV1. Therefore, it is not surprising that BCTC inhibited cough produced by aerosolized capsaicin exposure to the airways. We sort to examine the antitussive effects of BCTC in an ovalbumin sensitized guinea pig model. We found that BCTC and capsazepine suppressed antigen-evoked cough in the ovalbumin sensitized guinea pigs. Previous work by Bolser et al., (1995) demonstrated that allergic guinea pig could be used to characterize the cough suppressant activity of different pharmacological classes of antitussive drugs, including opioids, such as codeine [20]. Two defining features of the allergic guinea pig model are respiratory inflammation (mainly eosinophilia) and a hyperresponsiveness to pulmonary constricting agents such as histamine and methacholine [31]. It is becoming increasingly evident that pulmonary inflammation alters the excitability of afferent airway nerves that are important in the initiation of cough [18,32]. However, the mechanism(s) by which the excitability of sensory nerves is increased after inflammation is not completely established. Nevertheless, several studies have demonstrated that allergic inflammation significantly enhances the expression of tachykinins (SP and NKA) and tachykinin receptors (NK_2 _subtype) in vagal nodose ganglia [18,33,34]. It is also possible that chronic inflammation may enhance the functionality of afferent cough nerves at the level of the TRPV1 receptor. The sensitivity of capsaicin-induced cough responses following upper respiratory tract infection and in airway inflammatory diseases such as asthma and COPD, is increased relative to control responses [22,23]. Our findings in conjunction with above mentioned studies strongly support the position that TRPV1 is an attractive pharmacological target for the development of new antitussive drugs. Moreover, TRPV1 may have an increasing relevance as a target in respiratory diseases as inflammation becomes progressively chronic.

An important characteristic of the allergic guinea pig is that pulmonary exposure of antigen can produce an acute bronchoconstriction. The extent to which bronchoconstriction contributes to cough responses in the present model is not clear. It should be pointed out that bronchoconstriction and cough are not necessarily linked occurrences and may be mediated by different mechanisms [35]. In support of this hypothesis, we have found that when a prominent mast cell mediator, histamine (0.01%), is aerosolized to conscious naive guinea pigs it produces a 700% increase in a, Penh (a surrogate marker of bronchoconstriction; data not shown). On the other hand, this same concentration of histamine does not elicit cough. Nonetheless, studies to determine the extent to which BCTC and capsazepine attenuates antigen-evoked bronchoconstriction is beyond the scope of this report. This report focuses solely on TRPV1 blockade and antigen mediated tussigenic responses.

In summary, the data from this study show that TRPV1 antagonists inhibit cough elicited by aerosol exposure of ovalbumin in sensitized guinea pigs. The present study suggests that TRPV1 may play an important role in inflammatory cough. Specifically, in cough associated with pulmonary inflammation, such as found in some asthmatic patients.
